# Single and Combined Effects of *Clostridium butyricum* and Coccidiosis Vaccine on Growth Performance and the Intestinal Microbiome of Broiler Chickens

**DOI:** 10.3389/fmicb.2022.811428

**Published:** 2022-04-25

**Authors:** Haiming Cai, Shenquan Liao, Juan Li, Qihong Liu, Shengjun Luo, Minna Lv, Xuhui Lin, Junjing Hu, Jianfei Zhang, Nanshan Qi, Mingfei Sun

**Affiliations:** ^1^Zhaoqing/Maoming Branch Center of Guangdong Laboratory for Lingnan Modern Agricultural Science and Technology, Key Laboratory of Livestock Disease Prevention of Guangdong Province, Key Laboratory of Avian Influenza and Other Major Poultry Diseases Prevention and Control, Ministry of Agriculture and Rural Affairs, Institute of Animal Health, Guangdong Academy of Agricultural Sciences, Guangzhou, China; ^2^Jiangsu HFQ Biotechnology Co., Ltd., Haimen, China; ^3^Guangdong Qianyan Animal Health Care Co., Ltd, Guangzhou, China

**Keywords:** *Clostridium butyricum*, coccidiosis vaccine, production performance, intestinal microbiota, 16S rDNA

## Abstract

Avian coccidiosis is an important intestinal protozoan disease that has caused major economic losses to the poultry industry. *Clostridium butyricum* can not only maintain the stability of the intestinal barrier, but can also improve the production performance of broiler chickens. We studied the effects of feeding *C. butyricum* alone, administration of coccidiosis vaccine alone, and the combined administration of *C. butyricum* and coccidiosis vaccine on body weight gain, feed consumption, and feed conversion ratio of broilers. Meanwhile, intestinal contents of 8- and 15-day-old broilers were collected, and their intestinal microbiome was characterized by high-throughput sequencing of the V3–V4 region of 16S rDNA. We analyzed the oocysts per gram values and lesion scores in the *C. butyricum* alone group, in a group challenged with the coccidiosis-causing parasite, *Eimeria*, and in groups simultaneously challenged *Eimeria* and pretreated with *C. butyricum*, the coccidiosis vaccine, or combined *C. butyricum* and coccidiosis vaccine. Intestinal tissue samples were collected from 32-day-old broilers for microbiome analysis. Our results showed that combination of *C. butyricum* with coccidiosis vaccine significantly improved the performance of broiler chickens and also significantly reduced the oocysts per gram value and intestinal lesions caused by *Eimeria* sp. infection. Furthermore, *C. butyricum* and coccidiosis vaccine administered alone or in combination significantly increased the relative abundance of the immune biomarker genus *Barnesiella*. The significant increase in the abundance of the Clostridia_UCG.014, *Eubacterium coprostanoligenes* group and *Bacteroides* was a key factor in controlling *Eimeria* sp. infection.

## Introduction

Avian coccidiosis is an intestinal disease caused by *Eimeri*a sp., which invade and infect the chicken digestive system. Avian coccidiosis seriously damages the economy of the chicken industry, causing impaired development, reduced egg production, and high rates of mortality (Quiroz-Castañeda and Dantán-González, 2015). It is estimated that the annual loss caused by avian coccidiosis to the domestic poultry industry is more than US$ 3 billion (Noack et al., 2019). At present, there are seven main species of *Eimeri*a sp. in chickens, among which *E. tenella*, *E. necatrix*, *E. maxima*, and *E. acervulina* are considered to be the most important parasites causing economic losses (Fatoba and Adeleke, 2018). Anti-coccidial drugs, such as monensin, maduramycin, and hainanmycin, have been developed and applied. However, since the 1970s, the emergence of drug-resistant avian coccidia has been far faster than the development of new drugs (Noack et al., 2019). At the same time, at present, drug use inevitably leads to an increase in drug residues in poultry meat, which has aroused public health concerns (Karmi, 2014). There is, therefore, an urgent need for alternative strategies to prevent and control avian coccidiosis. At present, the use of coccidiosis vaccine to control avian coccidiosis is a reliable method. However, this immunization method can also lead to a decline in early growth performance and may increase the susceptibility of chicks to secondary infections, such as necrotizing enteritis (Stringfellow et al., 2011; Ritzi et al., 2016). Therefore, reduction or elimination of the negative effects of live vaccines is an urgent issue to be addressed in the prevention and control of avian coccidiosis.

Intestinal microbiomes exist in all animals and are key in the treatment of intestinal diseases. Although intestinal microbiota are highly diverse and there might be significant differences between individuals, the core microbiota are conserved and have a high degree of functional redundancy (Barnes et al., 2020). Intestinal microbiota are essential for host nutrient absorption, intestinal structure regulation, resisting pathogen invasion, and promoting immune system development (Belkaid and Hand, 2014). In many diseases, such as obesity (Al-Assal et al., 2018), inflammatory bowel disease (Khan et al., 2019), allergy (Pascal et al., 2018), antibiotic-associated diarrhea (Young and Schmidt, 2004), and opportunistic pathogen infection (Wallen et al., 2020), there is a correlation between microbiome changes and disease status. However, the intake of probiotics, such as *Lactobacillus*, *Lactococcus*, or *Bifidobacterium*, has little impact on the microbial population composition of healthy individuals, but it can affect the microbial population composition of diseased individuals, inhibiting the breeding of pathogenic bacteria and conditional pathogenic bacteria and improving intestinal health (Dhama et al., 2011; Agazzi, 2015; Mu et al., 2018). Previously, Chen et al. found that among the intestinal microbiota of chickens infected by *E. tenella*, the probiotics, *Lactobacillus*, *Faecalibacterium*, *Ruminococcaceae* UCG-013, *Romboutsia*, and *Shuttleworthia* were decreased in abundance (Chen et al., 2020b). Therefore, avian coccidia might also aggravate the degree of host inflammation through the regulation of intestinal microbiota. Dalloul et al. (2005) found that probiotics can reduce intestinal inflammation and also reduce the number of fecal oocysts. However, it is still unclear whether this involves the restoration of intestinal microbiota diversity.

*Clostridium butyricum* is a gram-positive anaerobic bacterium that is found in the intestines of healthy animals and humans (Shimbo et al., 2005). *Clostridium butyricum* can inhibit the growth of pathogenic microorganisms by producing butyric acid, which is beneficial to the balance of intestinal microbiota (Chen et al., 2020a). In addition, *C. butyricum* is also used as a probiotic for the treatment of inflammatory bowel disease (Xie et al., 2020) and necrotic enteritis (Huang et al., 2019). However, the effect of *C. butyricum* on avian coccidia infection and the effect of *C. butyricum* combined with live attenuated vaccine on intestinal microbiota are still unclear. The purpose of this study was to determine changes in growth performance, immune function, and intestinal microbiota diversity of broilers treated with *C. butyricum* alone or in combination with live attenuated vaccine. Changes in intestinal microbiota diversity of broilers infected with avian coccidia under the above preventive treatment were also assessed.

## Materials and Methods

### Materials Preparation

One-day-old Mahuang broilers were purchased from the farm in Chaozhou city; the room and cages were sterilized using blaze disinfection gun to obtain coccidian-free environment. The daily feed was 102 completed broiler feed produced by Shantou Wenfa Feed Industry Co., Ltd. The feed did not contain anti-coccidia drugs or antibiotics. Feed and water were available *ad libitum*. The chickens were randomly divided into four groups, three pens in each group, and separated drinker and feeder each pen. Four groups were separated from different rooms: 270 in control group, 90 chickens each pen, and 240 in each of three treatment groups, 80 chickens each pen. All the animal procedures were performed in accordance with the guidelines of the Animal Ethics Committee of Institute of Animal Health, Guangdong Academy of Agricultural Sciences.

The chicken coccidia strains used in this experiment are preserved at Institute of Animal Health, Guangdong Academy of Agricultural Sciences. The sporulation and purification of avian coccidia oocysts were performed as described previously (Ryley et al., 1976; Amer et al., 2010). The avian coccidiosis tetravalent live vaccine (*E. tenella* ETGZ strain, *E. necatrix* ENHZ strain, *E. acervulina* EAGZ strain, and *E. maxima* EMPY strain) was prepared by the Institute of animal health, Guangdong Academy of Agricultural Sciences. And the live vaccine was also prepared sporulated oocysts as above. *Clostridium butyricum* (2.0 × 10^8^ CFU/g) was purchased from Agricultural Science Group Qianyan Animal Health Co., Ltd. (Guangzhou, China).

### Experimental Design and Sample Collection

**Figure 1:**
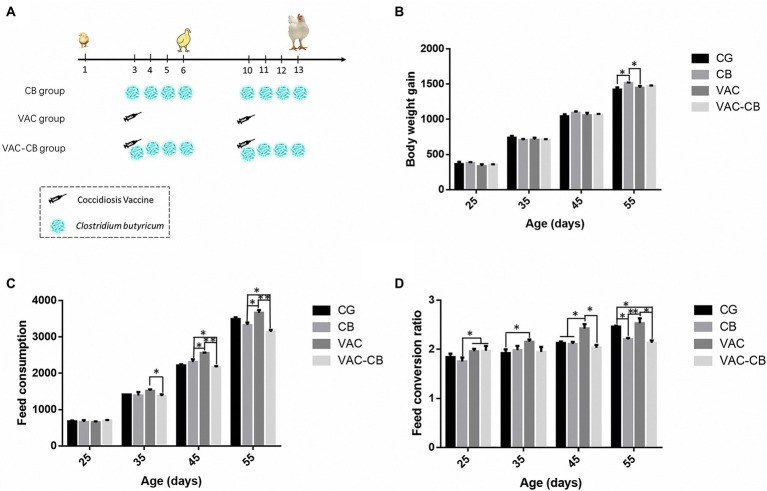
Technical roadmap **(A)** and production performance of Mahuang broilers administered *Clostridium butyricum* and coccidiosis vaccine. The production performance results include body weight gain **(B)**, feed consumption **(C)**, and feed conversion ratio **(D)**. The four experimental groups included control (CG), *C. butyricum* pretreatment (CB), coccidial vaccine pretreatment (VAC) and coccidial vaccine and *C. butyricum* co-pretreatment (VAC-CB) groups. Significant differences between groups are shown by asterisks: ^*^p < 0.05; ^**^p < 0.01.

The four groups included a control group (CG group), a *C. butyricum* pretreatment group (CB group), a vaccine pretreatment group (VAC group), and a vaccine and *C. butyricum* co-pretreatment group (VAC-CB group; Figure 1A). The VAC and VAC-CB groups were inoculated with 1,700 coccidiosis vaccine *via* drinking water with the sodium carboxymethyl cellulose (CMC, Dai-ichi Kogyo Seiyaku Co., Ltd. 0.0625% m/V) at the age of day 3, and the same dose of suspension was provided for the control group and CB group at the same time. They stopped drinking for 4 h before vaccine administration to ensure that chickens received the same dose of vaccine. Thereafter, the CB and VAC-CB groups were administered *C. butyricum* at 0.2 g/kg at the age of 3–6 days and 10–13 days, respectively. Five individuals were randomly taken from each group at the age of 8 and 15 days, and cecum contents were collected and snap-frozen in liquid nitrogen and stored at −80°C until DNA was isolated. In addition, body weight gain (BWG), feed consumption (FC), and feed conversion ratio (FCR) were determined at 1, 25, 35, 45, and 55 days old.

**Figure 2:**
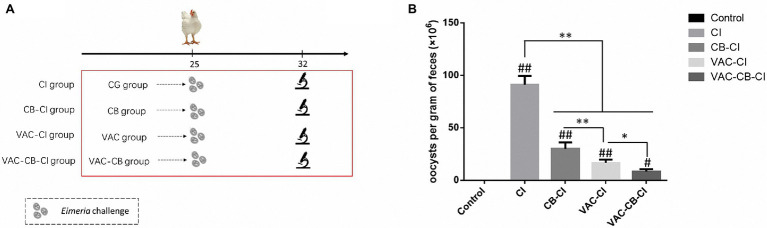
Technical roadmap **(A)** and oocysts per gram **(B)** of different pretreated chickens following *Eimeria* sp. challenge. The five experimental groups were CG, CI, CB-CI, VAC-CI, and VAC-CB-CI groups. The latter four groups were randomly taken from the CG, CB, VAC, and VAC-CB groups and then challenged with *Eimeria* sp. Significant differences between groups are shown by asterisks: ^*^p < 0.05; ^**^p < 0.01. Significant differences between treatment groups and the control group are indicated by the hash symbol: ^#^*p* < 0.05; ^##^*p* < 0.01.

To observe differences in resistance to avian coccidia and the change of intestinal microbiota after each pretreatment, we set up an *Eimeria* sp. (Figure 2A) infection treatment test on the basis of pretreatment grouping. At 25 days old, 30 chickens from all treatment groups were randomly infected with 34,000 chicken coccidia sporulated oocysts by oral administration. The chickens taken from CG, CB, VAC, and VAC-CB groups were assigned as coccidia infection group (CI group), CB-CI, VAC-CI, and VAC-CB-CI groups, respectively. In addition, another 30 chickens were taken from the CG group and assigned as a control group. The number of deaths, feed consumption, and weight changes were recorded daily, and then, BWG and FCR were calculated. The fecal samples were collected to calculate the number of oocysts per gram (OPG). At 32 days old, five individuals from each group were randomly taken and their cecum contents were collected. Pathological changes were observed, and the intestinal lesion score was determined (Ritzi et al., 2016). The intestinal tissues were then quickly frozen in liquid nitrogen and stored at −80°C for subsequent DNA extraction.

### DNA Extraction and 16S rRNA Sequencing

Metagenomic DNA was extracted from all cryopreserved intestinal tissue samples using an E.Z.N.A.® Stool DNA Kit according to the manufacturer’s instructions (Omega Bio-Tek, Norcross, GA, United States). The DNA samples were further purified using an Ultra Clean 15 DNA purification kit (MO BIO, Carlsbad, CA, United States), and the DNA concentration and quality were determined using a NanoDrop ND-1000 spectrophotometer (Thermo Scientific, Wilmington, Germany). Next, the primer pair (0.2 μM each of forward and reverse primers) 338F (5′-ACTCCTACGGGAGGCAGC AG-3′) and 806R (5′-GGACTACHVGGGTWTCTAAT-3′) was used to amplify the 16S rDNA V3–V4 hypervariable region. PCR conditions were initial denaturation at 94°C for 2 min, followed by 30 cycles of denaturation at 94°C for 30 s, annealing at 55°C for 30 s, extension at 72°C for 60 s, and finally, 72°C for 10 min. All PCR products were purified using a QIAquick PCR purification kit (Qiagen, Germany) and quantified with a NanoDrop ND-1000 spectrophotometer. Purified PCR products were quantified by Majorbio Bio-Pharm Technology Co., Ltd. (Shanghai, China), and sequencing was performed on an Illumina MiSeq paired-end (PE) 300 sequencing platform (Illumina Inc., United States).

### Bioinformatics Data Analysis

The Quantitative Insights into Microbial Ecology 2 (QIIME2) version 2020.11 pipeline was used to process the paired-end raw reads. q2-demux was used to demultiplex the raw double-ended reads. q2-cutadapt was used to remove primers and Illumina adapter sequences, and then, the Divisive Amplicon Denoising Algorithm (DADA2) plugin q2-dada2 was used to perform denoising of sequencing results and removal of chimeras. After denoising by dada2, representative sequences and an amplicon sequence variant (ASV) table of each sample were obtained. Then, taxonomic annotation of the ASV against the Silva database (138 release) at a 99% shared identity was performed using the QIIME2 feature-classifier classify-sklearn function. Otherwise, representative sequences were aligned using the qiime2 plugin phylogeny-to-tree-mafft-fasttree to build a rooted phylogenetic tree. Subsequent alpha and beta diversity comparisons were all done in RStudio version 1.4.1717. Rarefaction curve, observed taxa, chao1 index, Shannon diversity, and inverse Simpson diversity were performed by using R-package phyloseq v1.22.3. Otherwise, principal coordinate analysis (PCoA) with unweighted UniFrac and Bray–Curtis metrics were performed using the ordinate function, and plots were drawn using the plot_ordination function from the phyloseq package. In addition, linear discriminant analysis (LDA) and effect size (LEfSe) algorithms^1^ were used to identify significant microbial differences among groups. A significance value less than 0.05 and an LDA value greater than 4 were used as the threshold for LEfSe analysis. In addition, the DESeq2 package was used to complete the difference analysis of the relative abundance of microbial communities.

### Statistical Analysis

Repeated-measures ANOVAs were employed to compare experimental groups with time as a within-subject variable. Comparisons between experimental groups at a single time point were performed using one-way ANOVA followed by Tukey’s honest significant differences (HSD) *post hoc* test. All statistical analyses were performed using Social Scientists (SPSS) version 19 (SPSS Inc., United States) and GraphPad Prism version 6 (GraphPad, CA). The results are reported as the mean ± SEM. Significance was taken at a *p* value of <0.05.

## Results

### Growth, Feed Consumption, and FCR

At 55 days old, the weight gain of the CB group was significantly higher than that of the CG and VAC groups (*p* < 0.05; Figure 1B). At the same time, *C. butyricum* had greater effects on dietary consumption and feed conversion ratio in immunized broilers (Figures 1C,D). At 35 days old, compared with the VAC group, the VAC-CB group had a significantly reduced FC value (*p* < 0.05). Thereafter, at 45 and 55 days old, the average daily feed consumption of broilers after coccidiosis vaccine and *C. butyricum* co-pretreatment was 2,160.48 ± 22.74 g and 3,134.02 ± 35.19 g, while the corresponding results of the VAC group were 2,556.45 ± 9.05 g and 3,660.41 ± 44.10 g. Therefore, *C. butyricum* could significantly reduce the increase in dietary consumption caused by immunization. In addition, the FC value of the CB group was significantly lower than that of the VAC group (*p* < 0.05). The FCR value of the CB group was also significantly lower than that of the VAC and VAC-CB groups at 25 days old (*p* < 0.05). Compared with the VAC group, the VAC-CB and CB groups had significantly reduced FCRs at 45 and 55 days. Therefore, after two doses, *C. butyricum* had a significant impact on the production performance of broilers, especially on the recovery of production performance after immunization with chicken coccidia vaccine.

### Lesion Scores and OPG Following *Eimeria* sp. Challenge

To compare the preventive effects of different immunization and *C. butyricum* treatments on chicken coccidia infection, four treatment groups and a blank control were set up, and fecal oocysts analyzed. The OPG results are shown in Figure 2B. Compared with the control group, the OPG value of *Eimeria* sp. challenge alone was as high as 9.11 × 10^7^. However, different immunization and *C. butyricum* treatments significantly reduced the OPG value (*p* < 0.01), and the average OPG values of CB-CI and VAC-CI groups were 3 × 10^7^ and 1.65 × 10^7^, respectively. The OPG value of the VAC-CB-CI group (about 8.38 × 10^6^) was the lowest, indicating that the addition of probiotic feed significantly reduced the number of fecal oocysts (*p* < 0.05).

Scoring and statistical analysis were also performed for intestinal segment lesions in the experimental groups. The results are shown in Table 1. The lesion scores of cecum, jejunum, and duodenum of CI and CB-CI groups were significantly higher than that of VAC-CI, VAC-CB-CI, and control groups, there was no significant difference between CI and CB-CI groups, the lesion scores of cecum of CB-CI was significantly higher than that of CI group (*p* ≤ 0.05), the lesion scores of jejunum and duodenum of VAC-CB-CI group were significant lower than that of VAC-CI group. All intestinal segments of control group had no any lesions. Therefore, the combined administration of *C. butyricum* and chicken coccidiosis vaccine was of great significance to ensure intestinal health.

**Table 1:** Lesion score in various digestive organs isolated from treatment groups.

Group^a^	Cecum				Jejunum				Duodenum				*p*-Value^b^
	Means	SEM	Max	Min	Means	SEM	Max	Min	Means	SEM	Max	Min	
Control	0.00^D^	0.00	0	0	0.00^C^	0.00	0	0	0.00^C^	0.00	0	0	1.000
CI	2.04^A^	0.14	3	1	1.44^A^	0.14	2	0	1.40^A^	0.12	3	1	0.002
CB-CI	1.52^B^	0.10	2	1	1.40^A^	0.10	2	1	1.36^A^	0.10	2	1	0.465
VAC-CI	0.80^C^	0.12	2	0	0.68^B^	0.10	1	0	0.60^B^	0.10	1	0	0.288
VAC-CB-CI	0.56^C^	0.17	3	0	0.28^BC^	0.12	2	0	0.56^BC^	0.15	2	0	0.497

SEM, standard error of the mean. Means within a row with no common superscript letter (A–B) are significantly different (*p* ≤ 0.05).

a Group: Control, chicken without *Clostridium butyricum* and coccidiosis vaccine administration and *Eimeria* challenge; CI, chicken challenged with *Eimeria*; CB-CI, chicken fed with *C. butyricum* and also challenged with *Eimeria*; VAC-CI, chicken administrated with coccidiosis vaccine and challenged with *Eimeria*; VAC-CB-CI, chicken administrated with *C. butyricum* and coccidiosis vaccine, and then challenged with *Eimeria*.

b Overall treatment *p*-value.

### Sequencing Results and Quality Control

Eight-day-old and 15-day-old animals from CG, CB, VAC, and VAC-CB groups were selected for monitoring microbiota abundance. Four animals were randomly selected for intestinal sample collection. The V3–V4 region of 16S rDNA was sequenced, and the intestinal microbial composition of each group was evaluated. The sequencing included 32 samples, and a total of 3,619,574 raw reads were obtained. After quality filtering and chimerism detection by Qiime2, the pretrained Silva database (release 138, 99% ASV) was used for clustering and deleting all non-bacterial and low-frequency features. Finally, 628 ASVs were clustered at the same time and the coverage of samples from different groups was evaluated using rarefaction curves (Supplementary Figure S1), which showed that most samples were deeply sequenced.

We continued to study the chicken coccidia challenge in the immune regulation test group when the test animals reached 32 days old (i.e., 7 days after challenge). Four test animals were randomly selected for intestinal microbiota abundance monitoring. This test involved CON, CI, CB-CI, VAC-CI, and VAC-CB-CI groups. We obtained 2,146,476 raw reads through high-throughput sequencing, and 579 ASVs were obtained after Qiime2 cleaning and clustering. In addition, rarefaction curves (Supplementary Figure S2) indicated that most sequencing results had sufficient sequencing depth to meet the subsequent microbiome analysis.

### *Clostridium butyricum* and Coccidiosis Vaccination Change the Diversity of the Intestinal Microbiome

**Figure 3:**
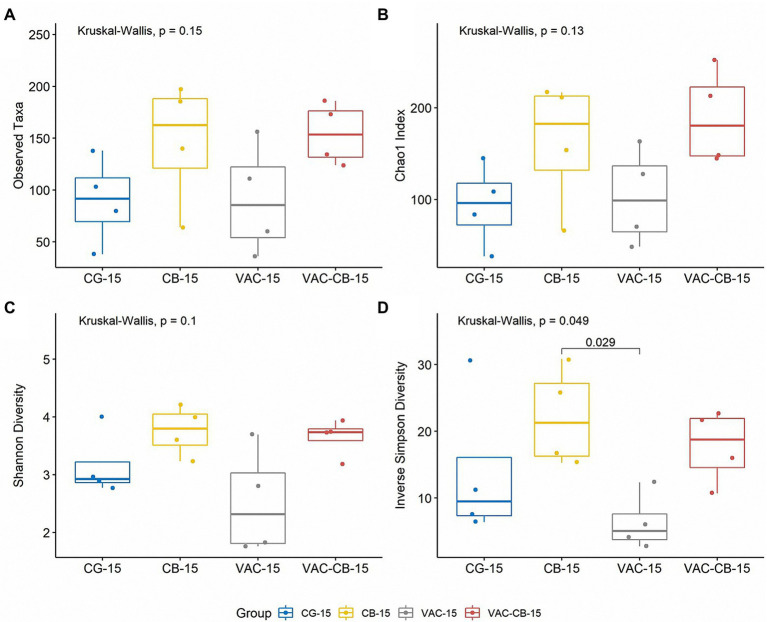
Comparison of intestine microbiome alpha diversity from various pretreatment groups at 15 days old. The alpha diversity indices including observed taxa **(A)**, chao1 index **(B)**, Shannon diversity **(C)**, and inverse Simpson diversity **(D)** were analyzed using the alpha function from the microbiome package. Boxplots were drawn using the ggboxplot function from the ggpubr package in R version 4.1.0. Alpha diversity was tested using the Kruskal–Wallis test with *post hoc* Dunn tests. The Wilcoxon test was performed to determine significant difference between groups. The box plots show the median, and whiskers show 25 and 75% quartiles.

To evaluate the effect of oral administration of *C. butyricum* and coccidiosis vaccine on alpha diversity of the intestinal microbiome, we recorded the observed taxa, Chao1 index, Shannon diversity, and inverse Simpson diversity of each sample. When a comparison was made between the test groups at 8 days old and 15 days old, although the diversity was slightly different at different ages, the difference was not statistically significant (Kruskal–Wallis test; *p* > 0.05; Supplementary Figure S3). However, at 15 days old, compared with the alpha diversity of all test groups, the observed taxa and Chao1 indexes of all test groups (CB group and VAC-CB group) fed with *C. butyricum* were greater than those of the control and VAC groups, indicating that *C. butyricum* increased the richness of the microbial community (Figures 3A,B). In addition, Shannon diversity and Simpson diversity were similarly high (Figures 3C,D). There was a significant difference in Simpson diversity between CB and VAC groups at 15 days old (Wilcoxon test; *p* ≤ 0.05; Figure 3D). Therefore, *C. butyricum* increased the community evenness of intestinal microbiota.

**Figure 4:**
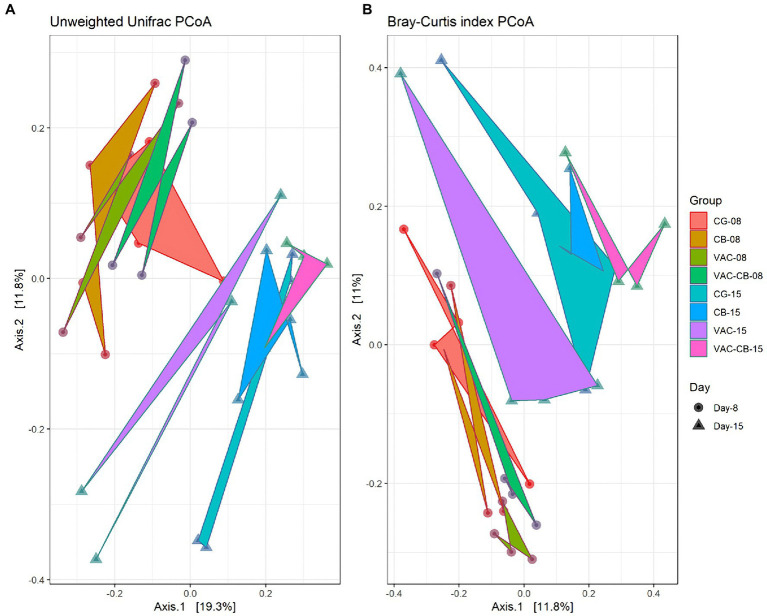
Beta diversity assessment of the intestine microbiome in various pretreatment groups at 8 and 15 days old. Three-dimensional scatter plots were generated using principal coordinates analysis (PCoA). PCoA was performed using ordinate function, and plots were drawn using the plot_ordination function from the phyloseq package. Distances shown in the PCoA plot are based on unweighted UniFrac distances **(A)** and Bray–Curtis diversity metrics **(B)**, respectively. The bacterial microbiome of each sample is indicated with one dot. The different color blocks represent different experimental groups. The different traits of the dots represent the different ages of the experimental animals.

To clarify the distance matrix of intestine microbiomes between the test groups, we calculated β diversity. These distances were analyzed based on unweighted UniFrac metric and Bray–Curtis diversity metrics and are displayed in a principal coordinate analysis plot. The unweighted UniFrac plot (Figure 4A) showed that the 8-day-old test group and the 15-day-old test group based on PCoA axes 1 and 2 can be clearly distinguished, and there is no sample overlap between the VAC group and other groups at 15 days old. At the same time, a Bray–Curtis plot (Figure 4B) could clearly see the above separation characteristics. However, the microbiota of CB and VAC-CB groups partially overlap, indicating that the general composition of the microbiota of animals fed with *C. butyricum* or *C. butyricum* combined with the chicken coccidiosis vaccine was similar. Therefore, *C. butyricum* played an important role in the formation of the intestinal microbiota community structure.

### Microbiome Composition Is Regulated by *Clostridium butyricum* and Coccidiosis Vaccine

**Figure 5:**
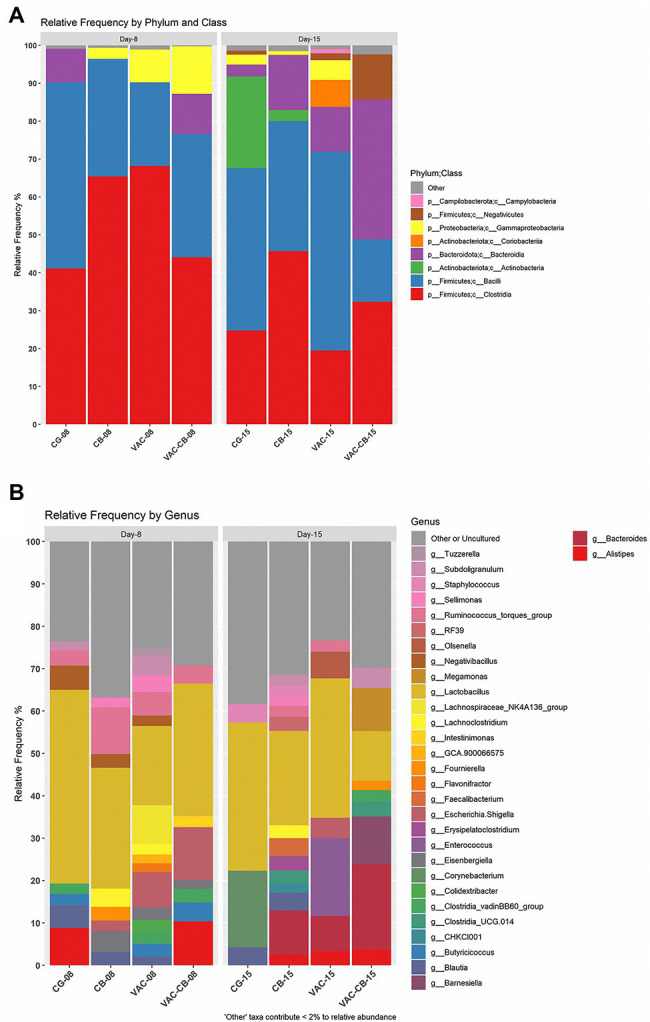
Relative abundance of intestine microbiome taxa of various pretreatment groups at 8 and 15 days old. The relative abundance of microbial families was analyzed for the different experimental groups at two classification levels, class **(A)** and genus **(B)**, and visualized by stacked bar plots. The analysis was performed using microbiome package and ggplot2 package in R version 4.1.0. At the class classification level, the taxa with an overall abundance of less than 1% were summed into “other.” At the genus classification level, the overall abundance was less than 2% and “uncultured” is classified as “other or uncultured.”

To clarify the effects of *C. butyricum* and live chicken coccidiosis vaccine on the composition of intestinal microbiota, the relative abundance of microbial taxa of the integral microbiome was analyzed. At the phylum level, *Firmicutes*, *Bacteroidota*, *Proteobacteria*, and *Actinobacteriata* were dominant in the intestinal microbiota of the different groups (Figure 5A). *Firmicutes* was the taxon with the highest relative abundance. The median abundance values of CG, CB, VAC, and VAC-CB groups were 90.29, 96.44, 90.26, and 76.63%, respectively, at 8 days of age, while they were 68.61, 80.78, 73.72, and 60.67%, respectively, at 15 days of age. At class level, *Clostridia*, *Bacilli*, *Bacteroidia*, and *Actinobacteria* were the main taxons (Figure 5A). The changes in the relative abundance levels of clostridia and bacilli may be closely related to treatment with *C. butyricum* and coccidiosis vaccine. At 8 days of age, the relative abundance of clostridia in VAC, CB, and VAC-CB groups was the highest, at 68.17, 65.38, and 44.07%, respectively. At this age, the highest relative abundance of *Bacilli* was in the CG group, which was 49.18%. However, at 15 days old, VAC and CG groups had the highest relative abundance of bacilli, at 52.44 and 42.83%, respectively, while CB and VAC-CB groups had the highest relative abundance of *Clostridia* and *Bacteroidia*, respectively. At the gene level (Figure 5B), *Lactobacillus* dominated in each experimental group. *Lactobacillus* had the highest relative abundance in CG, CB, VAC, and VAC-CB groups at 8 days old, accounting for 45.69, 28.50, 18.65, and 31.34%, respectively. However, *Lactobacillus* accounted for the highest proportion in CG, CB, and VAC groups at 15 days old, accounting for 34.95, 22.27, and 32.92%, respectively. The relative abundance of *Bacteroides* was highest in the VAC-CB group.

**Figure 6:**
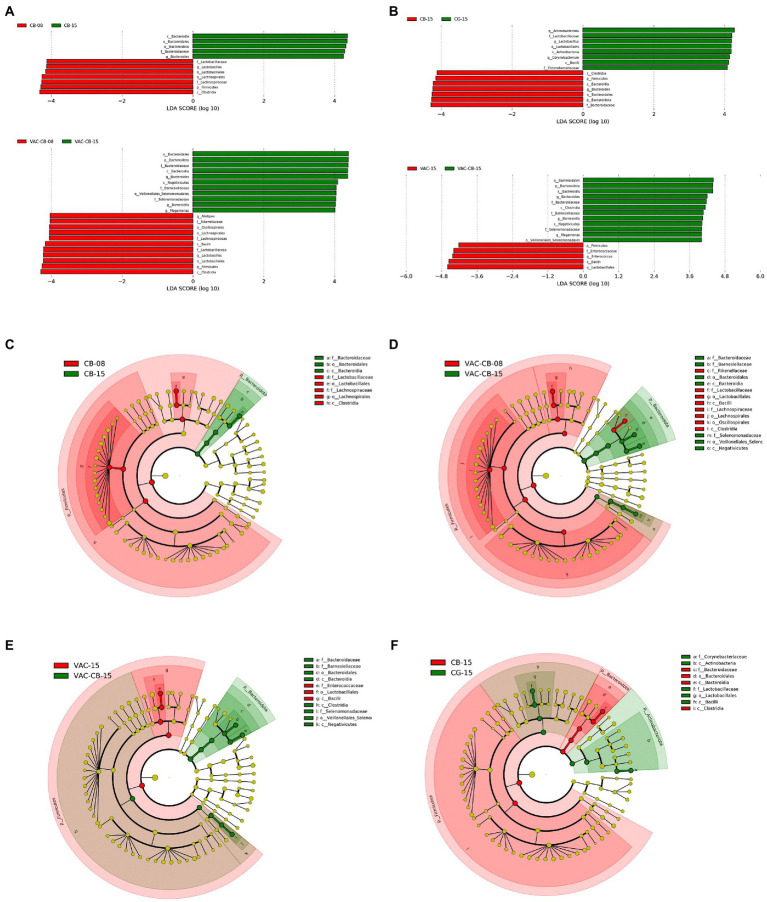
Linear discriminant analysis (LDA) effect size (LEfSe) for the intestine microbiome from various pretreatment groups at 8 and 15 days old. **(A,B)** Horizontal bars represent the effect size for each taxon. The length of the bar represents the log 10-transformed LDA score, indicated by vertical dotted lines. **(C–F)** Cladogram of the LEfSe analysis of the intestine microbiota among different groups. The threshold for the logarithmic LDA score was 4 and *p* < 0.05 for the factorial Kruskal–Wallis test among bacterial taxonomic units. The taxon level was abbreviated as p, phylum; c, class; o, order; f, family, and g, genus.

Through analysis of the dominant taxa, the differences in microbiota among groups could not be clearly explained. We further analyzed the impact of *C. butyricum* and coccidiosis vaccine on the intestinal microbiota of experimental animals using LEfSe analysis. We compared the increase or decrease in the relative abundance of the intestinal microbial taxa of 8-day-old and 15-day-old chickens among groups. The CB group showed important effects in these two time periods (Figures 6A,C). The phylum *Bacteroidota* played an important role in the 15-day-old chickens, which increased by 4.32-fold (*p* = 0.020) compared with the 8-day-old chickens. At the same time, the class *Bacteroidia* (*p* = 0.020), family *Bacterodaceae* (*p* = 0.014), and genus *Bacteroides* (*p* = 0.014) in the 15-day-old CB group were also fourfold more abundant than at the 8-day-old stage. The CB group is dominated by the phylum *Firmicute* at 8 days old and is fourfold higher than at 15 days old for the order *Lactobacillales*, family *Lactobacillaceae* and genus *Lactobacillus*. Correspondingly, the difference analysis results of the VAC-CB group’s bacterial microbiota at 8 and 15 days (Figures 6A,D) were similar to those of the CB group. Phylum *Firmicute* and phylum *Bacteroidota* still dominate, but at 15 days old, the genus *Barnesiella* (*p* = 0.013) in the VAC-CB group was increased by 4.03-fold compared with that at 8 days old. Immediately afterward, we performed a bacterial population difference analysis on the 15-day-old test group. The phylum *Bacteroidota* was increased by 4.27-fold in the CB group compared with the CG group and was significantly different for the genus *Bacteroides* (*p* = 0.021; Figures 6B,F). In addition, compared with VAC group, the VAC-CB group had high abundance of the genera *Bacteroidota* (4.20-fold), *Barnesiella* (4.05-fold), and *Megamonas* (4.00-fold, Figures 6A,E). Therefore, the results showed that whether the experimental animals were immunized with live chicken coccidiosis vaccine or not, as long as they were fed *C. butyricum*, the dominant species of the gut microbiome were similar, and phylum *Bacteroidota* was significantly increased.

### Microbial Diversity Changes in Chickens Pretreated With *Clostridium butyricum* and Coccidiosis Vaccine and Then Challenged With *Eimeria* sp.

To further explore the effect of *C. butyricum* and coccidiosis vaccine alone and in combination on the prevention and control of microbial diversity of *Eimeria* sp. infection, we conducted alpha diversity and beta diversity analysis at the same time. This test mainly involved five experimental groups: CON, CI, CB-CI, VAC-CI, and VAC-CB-CI groups. There were four samples in each experimental group. We calculated and analyzed the observed taxa, Chao1 index, Shannon diversity, and inverse Simpson diversity for each group. All groups had only slight differences in alpha diversity, and the differences were not statistically significant (Kruskal–Wallis test; *p* > 0.05, Supplementary Figure S4). Therefore, *Eimeria* infection had no measurable effect on alpha diversity, and the uniformity and abundance of the microbial community were similar in each group.

**Figure 7:**
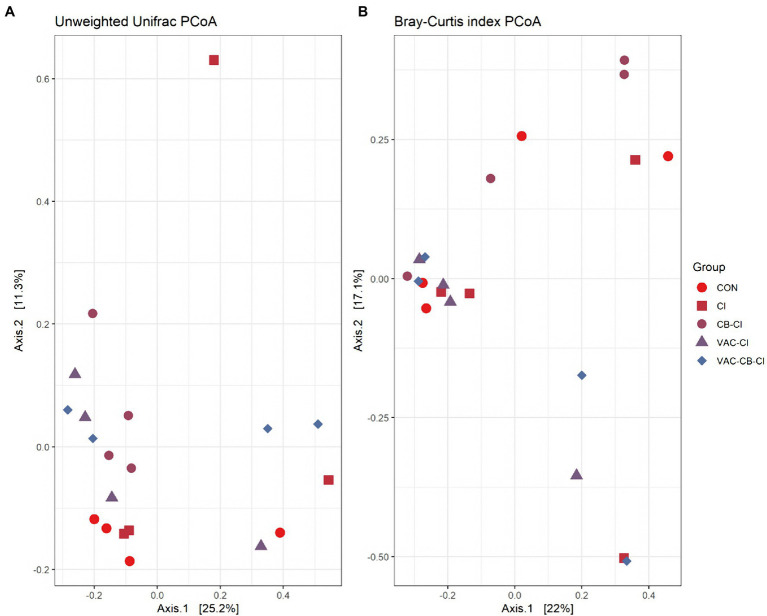
Beta diversity assessment of the intestine microbiome from different samples pretreated with *Clostridium butyricum* and coccidiosis vaccine and then challenged with *Eimeria* sp. PCoA plots of unweighted UniFrac distances **(A)** and Bray–Curtis distances **(B)** between samples were drawn using phyloseq package. The bacterial microbiome of each sample is indicated with one dot. The color blocks of different color traits represent different experimental groups.

We also evaluated the similarity between groups and samples of the test group using a PCoA plot (Figure 7) and a correlation Heatmap (Supplementary Figure S5). The PCoA plot was analyzed with unweighted UniFrac metrics (Figure 7A) and Bray–Curtis diversity metrics (Figure 7B). The unweighted UniFrac PCoA plot showed that the samples of most test groups were separated in the coordinate system, but that the sample dispersion of the CI group was large, and there may be differences in the general composition of microbial taxa. Combined with the Bray–Curtis PCoA plot, we found significant overlap among CON, VAC-CB-CI, and VAC-CI groups, indicating that the general composition of the microbial communities in these groups may be similar. The cluster analysis of each sample based on taxon composition was further combined with a correlation plot (Supplementary Figure S5). This showed that except for individual sample deviation, most samples have good similarity at the same grouping level.

### *Eimeria* Infection Regulated the Intestine Microbial Community Induced by *Clostridium butyricum* and the Coccidiosis Vaccine

**Figure 8:**
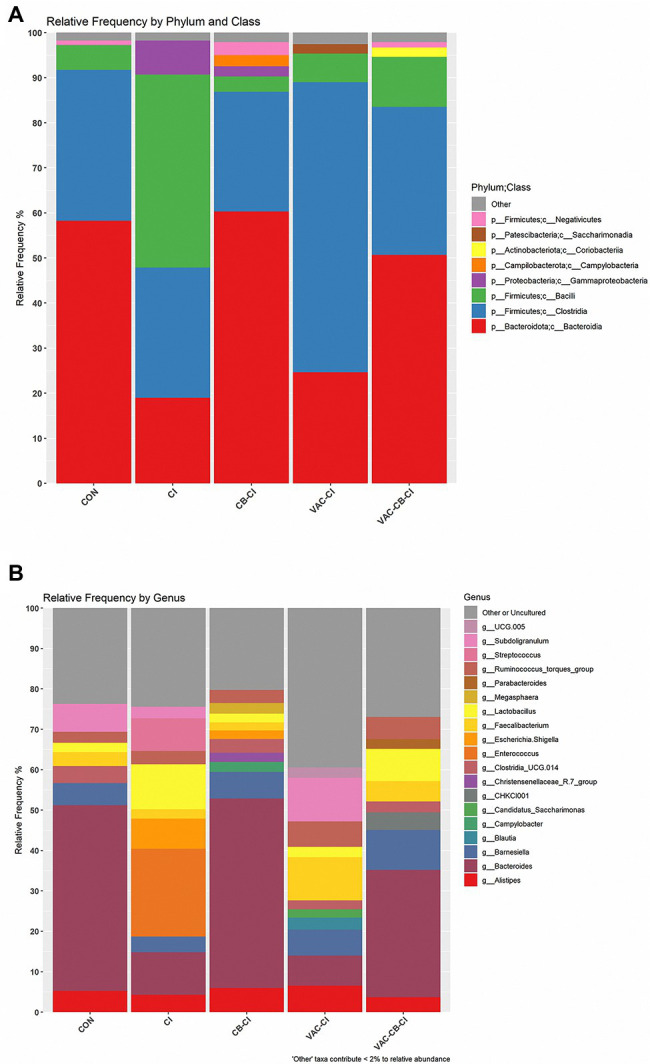
Relative abundance of intestine microbiome taxa from samples pretreated with *Clostridium butyricum* and coccidiosis vaccine and then challenged with *Eimeria* sp. The relative abundances of gut bacteria presented in the plot were calculated by averaging the data obtained from the four replicates within each group. The relative abundance of microbial taxa was performed at two classification levels, class **(A)** and genus **(B)**. At the class level, the taxa with an overall abundance of less than 1% were summed into “other.” At the genus level, the overall abundance was less than 2% and “uncultured” is classified as “other or uncultured.”

To clarify the impact of *Eimeria* sp. infection on the composition of microbiota, an integral micro-community analysis was conducted for each group at phylum, class, and genus levels. At phylum level, *Firmicutes* and *Bacteroidota* were taxa with the highest relative abundance (Figure 8A). Among them, the relative abundance of *Bacteroidota* was the highest in CON, CB-CI, and VAC-CB-CI groups at 58.22, 60.31, and 50.64%, respectively. The CI and VAC-CI groups were mainly enriched for phylum *Firmicutes*, at 71.81 and 70.92%, respectively. At class level, *Clostridia*, *Bacteroidia*, and *Bacilli* accounted for a large proportion (Figure 8A). The VAC-CI and CI groups were mainly *Clostridia* (64.37%) and *Bacilli* (42.77%). *Bacteroidia* was the high abundance taxon in CON, CB-CI, and VAC-CB-CI groups, with relative abundance of 58.22, 60.31, and 50.64%, respectively. At the gene level, *Bacteroides*, *Enterococcus*, and *Subdoligranulum* were high abundance taxa (Figure 8B). Among them, CON, CB-CI, and VAC-CB-CI groups had the highest relative abundance of genus *Bacteroides* at 46.00, 46.93, and 31.50%, respectively. The high abundance genus taxa of CI and VAC-CI groups were *Enterococcus* (27.74%) and *Subdoligranulum* (10.79%), respectively.

**Figure 9:**
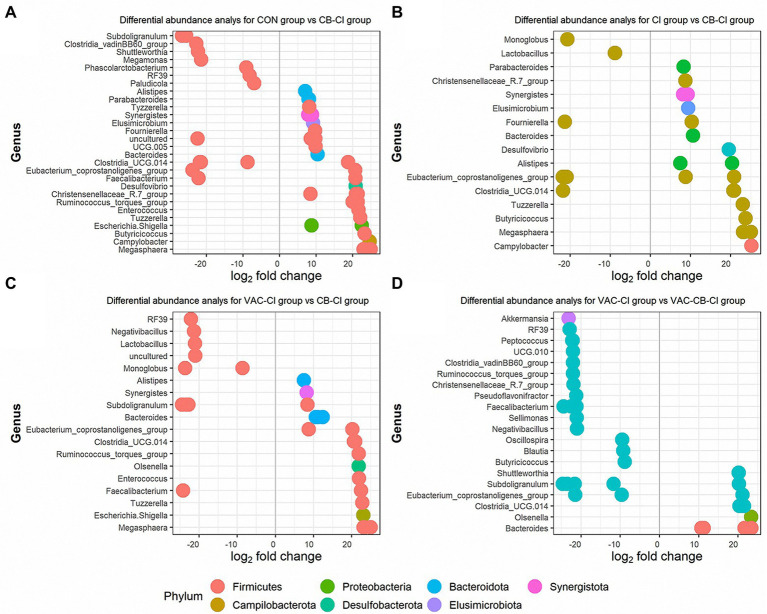
Differential abundance analysis at the level of operational taxonomic unit (OTU) of the intestine microbiome from samples pretreated with *Clostridium butyricum* and coccidiosis vaccine and then challenged with *Eimeria* sp. The *x*-axis represents the log2 (fold change) and the *y*-axis represents the OTU genus. Each colored dot represents a separate OTU, and the color represents a different phylum. The plot shows differentially abundant OTUs among different groups, including the CON group compared with the CB-CI group **(A)**, the CI group compared with the CB-CI group **(B)**, the VAC-CI group compared with the CB-CI group **(C)** and the VAC-CI group compared with the VAC-CB-CI group **(D)**. The analysis was performed using the DESeq2 package in R version 4.1.0.

We also used a DESeq2 method to further clarify the differential expression of microbiota in different test groups. We compared the differences in microbiota between CB-CI and CON groups, CI and VAC-CI groups, and between VAC-CB-CI and VAC-CI groups. Thirty ASVs and 14 ASVs were significantly increased in the CB-CI group compared with the CON group (Figure 9A). The significantly increased ASVs were mainly distributed in phyla *Firmicutes* (19 ASVs), *Synergistata* (2 ASVs), *Bacteroidota* (4 ASVs), *Proteobacteria* (2 ASVs), *Desufobacteria* (1 ASVs), *Campilobacterota* (1 ASVs), and *Elusimicrobiota* (1 ASVs). ASV differential abundance analysis was performed for the CB-CI and CI groups (Figure 9B). Twenty-one ASVs were significantly increased, and nine were significantly decreased. Among them, highly expressed ASVs were concentrated in phyla *Firmicutes* (12 ASVs), *Bacteroidota* (four ASVs), *Synergistota* (two ASVs), *Desulfobacterota* (one ASV), *Campilobacterota* (one ASV), and *Elismicrobiota* (one ASV). The CB-CI and VAC-CI groups were also compared (Figure 9C). Differential analysis showed that the relative abundance of 21 ASVs increased significantly and that of 10 ASVs decreased significantly. These high abundance ASVs were concentrated in phyla *Firmicutes* (14 ASVs), *Bacteroidota* (four ASVs), *Synergistota* (one ASV), *Proteobacteria* (one ASV), and *Actinobacterita* (one ASV). Among the significant increases in microbiota abundance, difference analysis of CB-CI and CON groups, and CI and VAC-CI groups, showed that genera *Bacteroides*, Clostridia_UCG.014, *Eubacterium coprostanogenes* group, *Megasphaera*, *Synergists*, *Tuzzerella*, and *Alistipes* appeared simultaneously, which might comprise the representative genes dominating the microbiota function of the CB-CI group. We also analyzed the difference in ASV abundance between VAC-CB-CI and VAC-CI groups (Figure 9D). Eleven ASVs were significantly increased, and 24 were significantly decreased. The ASVs with significantly increased relative abundance were concentrated in phyla *Firmicutes* (six ASVs), *Bacteroidota* (four ASVs), and *Actinobacterio* (one ASV). At the genus classification level, the difference in abundance of ASVs between VAC-CB-CI vs. VAC-CI groups was compared by the principal component of CB-CI group, and *Clostridia*_UCG.014, *E. coprostanogenes* group, and *Bacteroides* were similarly abundant.

## Discussion

In this study, we evaluated single and combined effects of *C. butyricum* and coccidiosis vaccine on growth performance and the intestine microbial community of broiler chickens. The combined use of *C. butyricum* and coccidiosis vaccine can attenuate the side effects of chicken coccidia vaccine alone on the production performance of broilers such as BWG, FC, and FCR. Further, we also found that *C. butyricum* can modulate intestinal health by regulating the composition and relative abundance of intestinal microbiome.

Coccidiosis vaccine administration can effectively prevent chicken coccidiosis infection, but it also reduces the production performance of broilers (Das et al., 2021). Probiotics can improve the production performance of broilers. For example, Yang et al. explored the effects of different doses of *C. butyricum* on the production performance of Ling Nan yellow broiler chickens. They found that 3 × 10^7^ CFU *C. butyricum*/kg of diet could significantly improve the production performance of broilers (Liao et al., 2015). Ritzi et al. (2016) previously showed that feeding Poultrystar (a probiotics product) alone or combined with Immucox I (a coccidiosis vaccine) could improve chicken performance in Cobb-500 broilers. Similar to previous reports, we also found that Mahuang broilers fed *C. butyricum* alone or combined with coccidiosis vaccine could significantly increase the BWG value and reduce the FC and FCR values. In addition, our study showed that when *Eimeria* sp. challenge was performed on all pretreated animals, the OPG value could be significantly reduced by the application of *C. butyricum* alone, coccidiosis vaccine alone, or by the combined application of *C. butyricum* and coccidiosis vaccine. At the same time, while comparing the VAC-CB-CI group with the VAC-CI or CI group, the intestinal lesion score in jejunum and duodenum intestinal segments was significantly decreased of the VAC-CB-CI group (*p* < 0.05). Previously, Lee et al. (2010) reported that chicken feed with *Bacillus* reduced the intestinal lesions after *E. maxima* challenge. In addition, Ritzi et al. (2016) also found that the intestinal lesion score was significantly reduced when Poultrystar and Immucox I were administered at the same time. Therefore, artificial supplementation of probiotics may help to improve the homeostasis of the natural intestinal barrier, repair the slight intestinal disruption caused by coccidiosis vaccination, maintain the integrity of the intestinal epithelium, and enhance nutrient absorption and utilization, so as to obtain better production performance.

We also analyzed the effects of *C. butyricum* and coccidiosis vaccination on intestinal microbiota of broilers by 16S rRNA gene sequencing. *Clostridium butyricum* is a strictly anaerobic endospore-forming gram-positive bacterium that produces butyric acid. *Clostridium butyricum* inhibits the reproduction and transmission of pathogens in the host, so is considered as a potential substitute for antibiotics (Zhao et al., 2017). Previously, Huang et al. (2019) found that *C. butyricum* in the diet can increase the relative proportion of clostridia at different stages of necrotic enteritis. We also found that clostridia (65.38%) were the dominant intestinal microbiota at 8 and 15 days of age when *C. butyricum* was administered alone. When *C. butyricum* was combined with the coccidiosis vaccine, there was a high abundance of phylum *clostridia* at 8 days old, while *Bacteroidia* was abundant at 15 days old. We also used LEfse to analyze representative immune biomarkers, in which the relative abundance of genus *Bacteroides* in the CB group increased significantly compared with the control group, while the VAC-CB group had higher relative abundance of the genera *Bacteroidota*, *Barnesiella*, and *Megamonas* (4.00-fold) compared with the VAC group. Early studies showed that the higher relative abundance of *Bacteroides* indicated a healthy gut microbiota (Adewole and Akinyemi, 2021). The genus *Bacteroides* is highly adaptable and can adjust to the nutritional conditions of the intestinal environment. *Bacteroides* also play an important role in decomposing complex polysaccharides, such as starch and cellulose, into simpler compounds. Wang et al. (2017) found that treatments with various probiotics supplemented in the diet increased the abundance of *Bacteroides*. Zhu et al. (2020) also found that dietary heat-inactivated compound probiotics increased the relative abundance of *Bacteroides*. Therefore, the genus *Bacteroides* is a key factor in maintaining intestinal health by administration of *C. butyricum* or *C. butyricum* combined with vaccine.

We also monitored the effect of *Eimeria* sp. challenge on the intestinal microbial community of broilers after single or combined administration of *C. butyricum* and coccidiosis vaccine. Previously, Hongliang et al. reported that *E. tenella* infection can lead to decreases in *Lactobacillus*, *Faecalibacterium*, *Ruminococcaceae* UCG-013, *Romboutsia*, and *Shuttleworthia* in the cecal microbial community. However, opportunistic pathogens, such as *Enterococcus* and *Streptococcus*, increased in relative abundance (Chen et al., 2020b). Our study found that *Eimeria* sp. infection leads to high *Enterococcus* (27.74%) and *Subdoligranulum* (10.79%) abundance (Figure 8). However, when *C. butyricum* and coccidiosis vaccine were administered alone or in combination before *Eimeria* sp. infection, the probiotic genus *Bacteroides* became a high abundance taxon. Therefore, administration of *C. butyricum* and coccidiosis vaccine alone or in combination play an important regulatory role in the health of intestinal microbiota. We also used Deseq2 to analyze the difference in abundance of ASVs (Figure 9). The results showed that the application of *C. butyricum* and coccidiosis vaccine alone or in combination significantly increased the relative abundance of Clostridia_UCG.014, *E. coprostanogenes* group, and *Bacteroides* in intestinal microbial colonies. *Eubacterium coprostanogenes* group functions in biotransforming cholesterol into fecal prostaglandin, which can further affect the fat metabolism of the host (Benítez-Páez et al., 2020). Cholesterol has important physiological effects in animals. *Bacteroides* also play an important role in intestinal health. Therefore, increasing the relative abundance of Clostridia_UCG.014, *E. coprostanogenes* group, and *Bacteroides* is a possible mechanism by which the combined application of *C. butyricum* and vaccine helps to maintain the intestinal microbial community against *Eimeria* sp. infection.

## Conclusion

Our results show that the application of combination *C. butyricum* and coccidiosis vaccine can significantly improve the production performance of broilers, significantly reduce the OPG value caused by *Eimeria* sp. Infection, and reduce intestinal lesions. In addition, by analyzing the intestinal microbial community, we also found that *C. butyricum* and coccidiosis vaccine alone or in combination can significantly increase the relative abundance of genus *Barnesiella*. At the same time, following *Eimeria* sp. infection, it could also promote the relative abundance of genus *barnesiella* and significantly increase Clostridia_UCG.014, *E. coprostanogenes* group, and *Bacteroides* in the intestinal tract, to ensure the health of intestinal microbiota and the integrity of the protective barrier. Our study provides new mechanistic insight into the effect of combined application of probiotics and coccidiosis vaccine.

## Data Availability Statement

The datasets presented in this study can be found in online repositories. The names of the repository/repositories and accession number(s) can be found at: https://www.ncbi.nlm.nih.gov/bioproject/PRJNA770159.

## Ethics Statement

The animal study was reviewed and approved by Animal Ethics Committee of Institute of Animal Health, Guangdong Academy of Agricultural Sciences.

## Author Contributions

NQ and MS conceived and designed the research. HC and SL analyzed the data and wrote the manuscript. JL, ML, XL, and JH performed the experiments and analyzed the data. QL, SL, and JZ revised the manuscript. NQ supervised the project. All authors contributed to the article and approved the submitted version.

## Funding

This work was supported by Key Realm R&D Program of Guangdong Province (2020B0202080004 and 2020B0202090004), NSFC grants (31872460), Guangdong Basic and Applied Basic Research Foundation (2021A1515010521, 2021A1515012401, 2020A1515011575, 2019A1515010913, and 2021B1515120006), Science and Technology Plan Projects of Guangdong Province (2021B1212050021), Science and technology project of Heyuan (2021008), Science and technology project of Yunfu (2021040202, 2021020604, and 2021020607), Science and technology project of Guangzhou (202102080459), Special fund for scientific innovation strategy-onstruction of high level Academy of Agriculture Science (XTXM202202, 202110TD, 202122TD, R2020PY-JC001, R2019YJ-YB3010, R2020PY-JG013, R2020QD-048, and R2021PY-QY007). Guangdong Provincial special fund for modern Agriculture Industry Technology Innovation teams (2022KJ119).

## Conflict of Interest

QL is employed by Jiangsu HFQ Biotechnology Co., Ltd., Haimen. SL is employed by Guangdong Qianyan Animal Health Care Co., Ltd.

The remaining authors declare that the research was conducted in the absence of any commercial or financial relationships that could be construed as a potential conflict of interest.

## Publisher’s Note

All claims expressed in this article are solely those of the authors and do not necessarily represent those of their affiliated organizations, or those of the publisher, the editors and the reviewers. Any product that may be evaluated in this article, or claim that may be made by its manufacturer, is not guaranteed or endorsed by the publisher.

## Footnotes

^1^ http://huttenhower.sph.harvard.edu/galaxy/

## Supplementary Material

Supplementary Figure S1 Rarefaction analysis of V3/V4 pyrosequencing reads of the 16S rRNA gene in intestinal microbiota from *Clostridium butyricum* and coccidiosis vaccine-administered samples. Rarefaction curves were drawn using the ggrare function from the ranacapa package in R version 4.1.0. Abbreviations: CG-08, samples from the control group at 8 days old. VAC-08, samples from the VAC group at 8 days old; CB-08, samples from the CB group at 8 days old; VAC-CB-08, samples from the VAC-CB group at 8 days old. Correspondingly, CG-15, CB-15, VAC-15, and VAC-CB-15 represent samples collected from CG, CB, VAC, and VAC-CB groups at 15 days old, respectively.

Supplementary Figure S2 Rarefaction analysis of V3/V4 pyrosequencing reads of the 16S rRNA gene in fecal microbiota from samples pretreated with *Clostridium butyricum* and coccidiosis vaccine and then challenged with *Eimeria*. Rarefaction curves built using ggrare script in R version 4.1.0. Abbreviations: CON, samples from the CG group at 32 days old. CI, CB-CI, VAC-CI, and VAC-CB-CI represent the samples randomly taken from CG, CB, VAC, and VAC-CB groups and subjected to *Eimeria* challenge and then collected at 32 days old.

Supplementary Figure S3 Boxplots of pairwise comparison of alpha diversity indices of intestinal microbiota from various groups between 8 days old and 15 days old using Wilcoxon’s nonparametric test. The alpha diversity indices including observed taxa **(A)**, chao1 index **(B)**, Shannon diversity **(C)**, and inverse Simpson diversity **(D)** were analyzed using the alpha function from the microbiome package. And then boxplots were draw using the ggboxplot function from the ggpubr package in R version 4.1.0. The box plots show the median, and whiskers show 25 and 75% quartiles.

Supplementary Figure S4 Comparison of intestine microbiome alpha diversity from samples pretreated with *Clostridium butyricum* and coccidiosis vaccine and then challenged with *Eimeria* sp. The alpha diversity indices, including observed taxa **(A)**, chao1 index **(B)**, Shannon diversity **(C)**, and inverse Simpson diversity **(D)** were visualized by boxplots. Alpha diversity was tested using the Kruskal–Wallis test with *post hoc* Dunn tests. The box plots show the median, and whiskers show 25 and 75% quartiles.

Supplementary Figure S5 Correlation heatmap for the intestine microbiome from samples pretreated with *Clostridium butyricum* and coccidiosis vaccine and then challenged with *Eimeria* sp. Correlation heatmap shows sample similarity by clustering the samples with similar taxa. Yellow indicates a close correlation, while green indicates a more distant correlation in the data. The heatmap was produced using the heatmap.2 function from gplots package in R version 4.1.0.
