# Supplementary figures and images for: Single and Combined Effects of Clostridium butyricum and Coccidiosis Vaccine on Growth Performance and the Intestinal Microbiome of Broiler Chickens
Source: Front Microbiol. 2022 Apr 25;13:811428. doi: 10.3389/fmicb.2022.811428 (PMC9083122; doi:10.3389/fmicb.2022.811428)

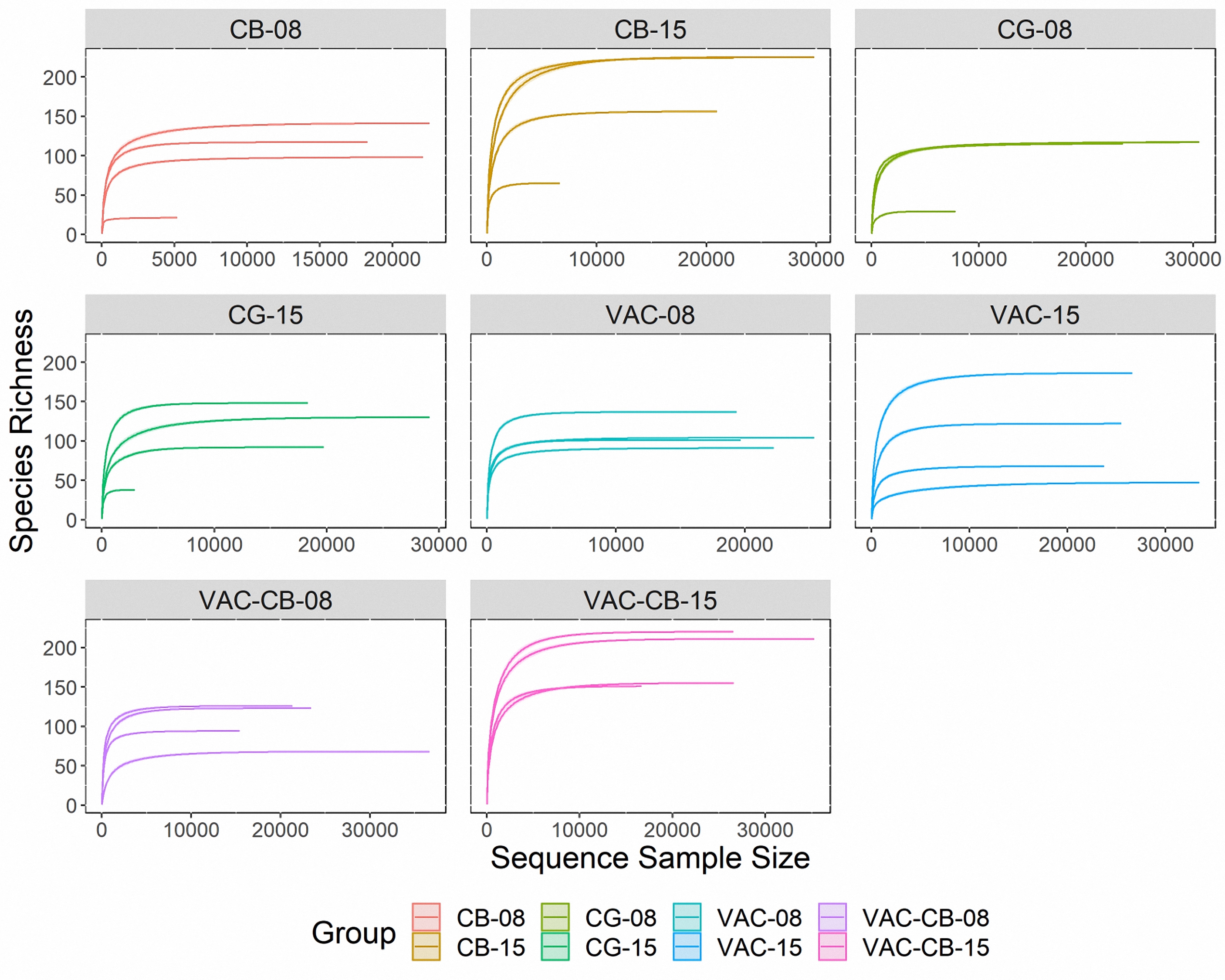

Supplement: Supplementary Figure S1 — Rarefaction analysis of V3/V4 pyrosequencing reads of the 16S rRNA gene in intestinal microbiota from Clostridium butyricum and coccidiosis vaccine-administered samples. Rarefaction curves were drawn using the ggrare function from the ranacapa package in R version 4.1.0. Abbreviations: CG-08, samples from the control group at 8 days old. VAC-08, samples from the VAC group at 8 days old; CB-08, samples from the CB group at 8 days old; VAC-CB-08, samples from the VAC-CB group at 8 days old. Correspondingly, CG-15, CB-15, VAC-15, and VAC-CB-15 represent samples collected from CG, CB, VAC, and VAC-CB groups at 15 days old, respectively. [file Image_1.JPEG]

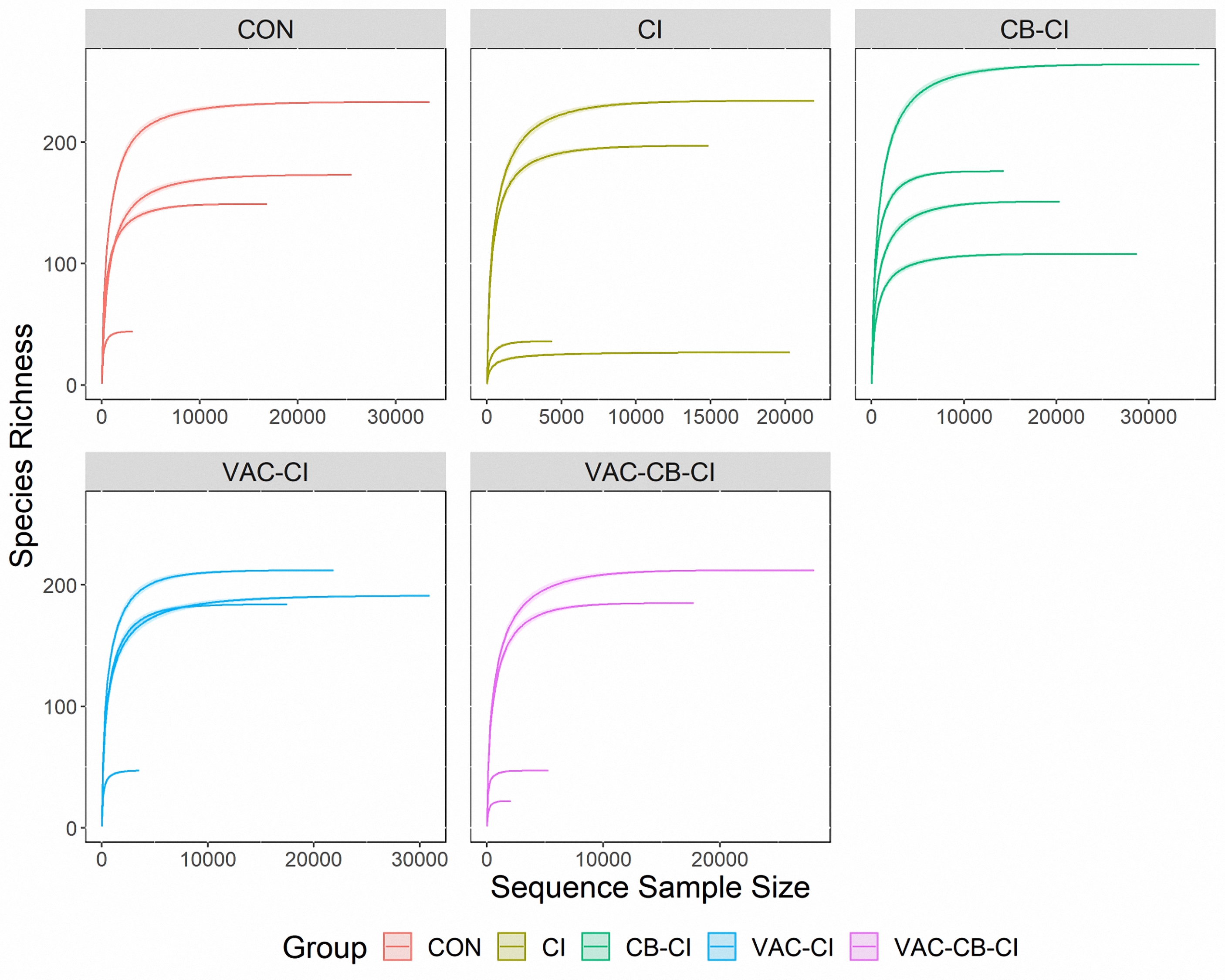

Supplement: Supplementary Figure S2 — Rarefaction analysis of V3/V4 pyrosequencing reads of the 16S rRNA gene in fecal microbiota from samples pretreated with Clostridium butyricum and coccidiosis vaccine and then challenged with Eimeria. Rarefaction curves built using ggrare script in R version 4.1.0. Abbreviations: CON, samples from the CG group at 32 days old. CI, CB-CI, VAC-CI, and VAC-CB-CI represent the samples randomly taken from CG, CB, VAC, and VAC-CB groups and subjected to Eimeria challenge and then collected at 32 days old. [file Image_2.JPEG]

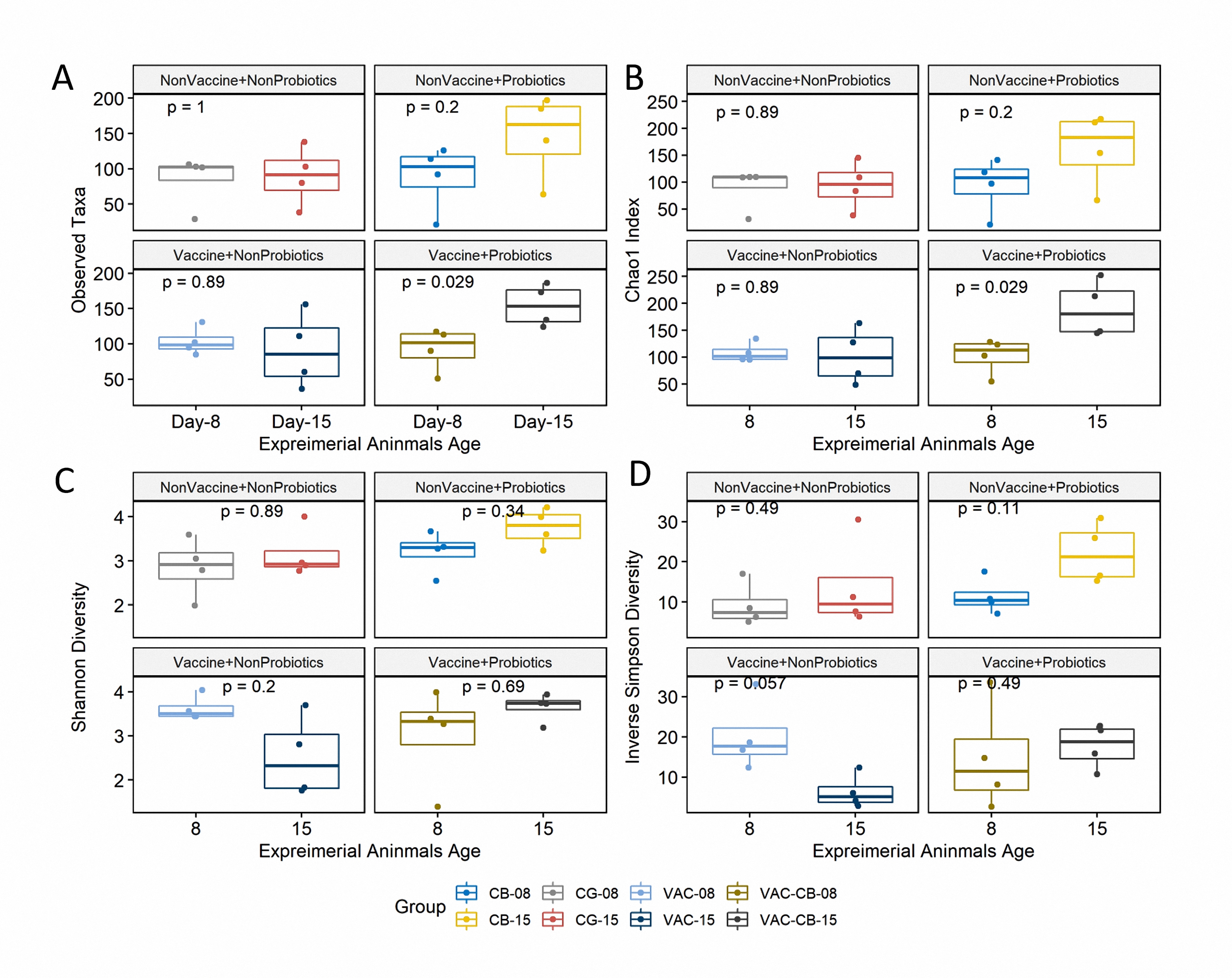

Supplement: Supplementary Figure S3 — Boxplots of pairwise comparison of alpha diversity indices of intestinal microbiota from various groups between 8 days old and 15 days old using Wilcoxon’s nonparametric test. The alpha diversity indices including observed taxa (A), chao1 index (B), Shannon diversity (C), and inverse Simpson diversity (D) were analyzed using the alpha function from the microbiome package. And then boxplots were draw using the ggboxplot function from the ggpubr package in R version 4.1.0. The box plots show the median, and whiskers show 25 and 75% quartiles. [file Image_3.JPEG]

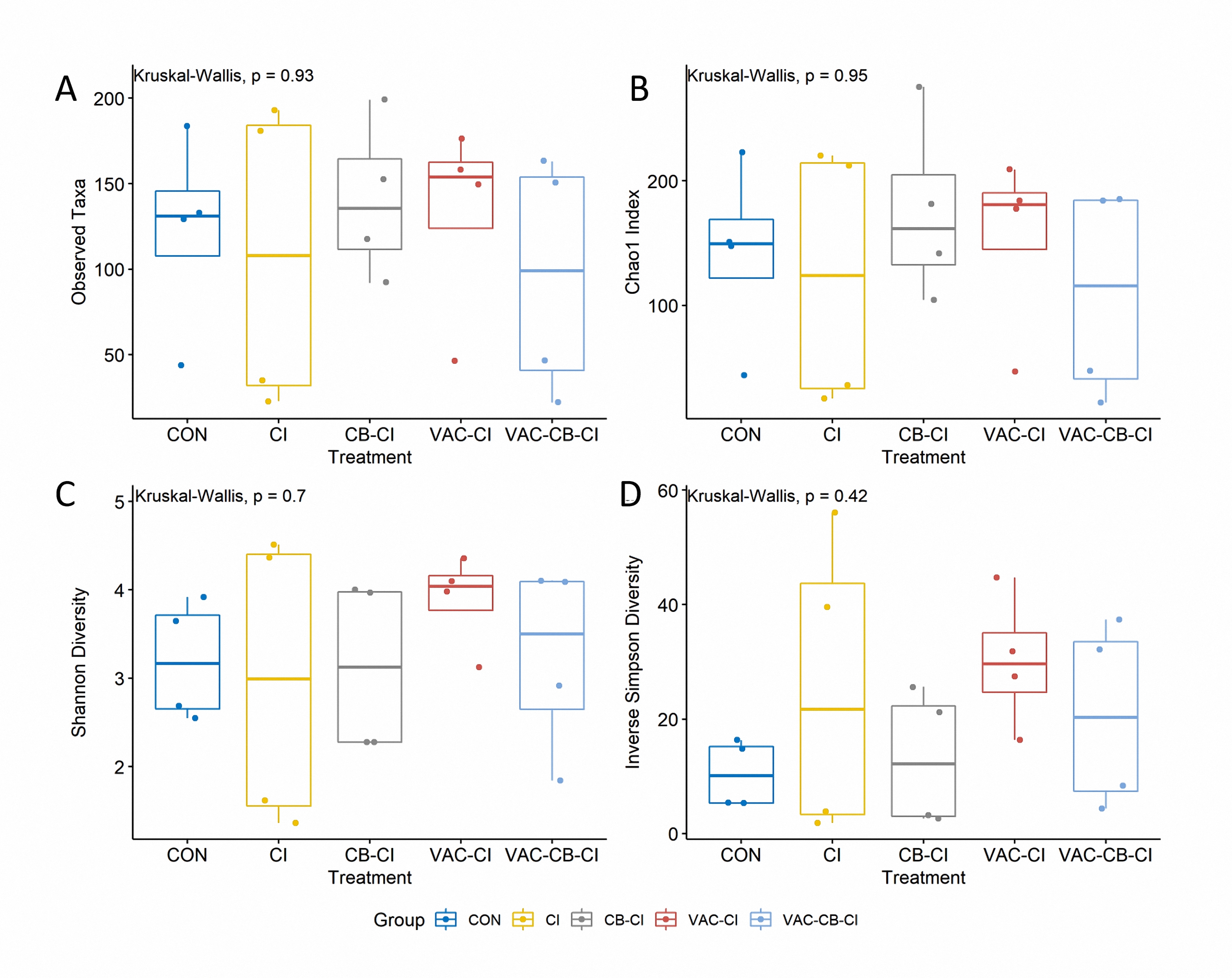

Supplement: Supplementary Figure S4 — Comparison of intestine microbiome alpha diversity from samples pretreated with Clostridium butyricum and coccidiosis vaccine and then challenged with Eimeria sp. The alpha diversity indices, including observed taxa (A), chao1 index (B), Shannon diversity (C), and inverse Simpson diversity (D) were visualized by boxplots. Alpha diversity was tested using the Kruskal–Wallis test with post hoc Dunn tests. The box plots show the median, and whiskers show 25 and 75% quartiles. [file Image_4.JPEG]

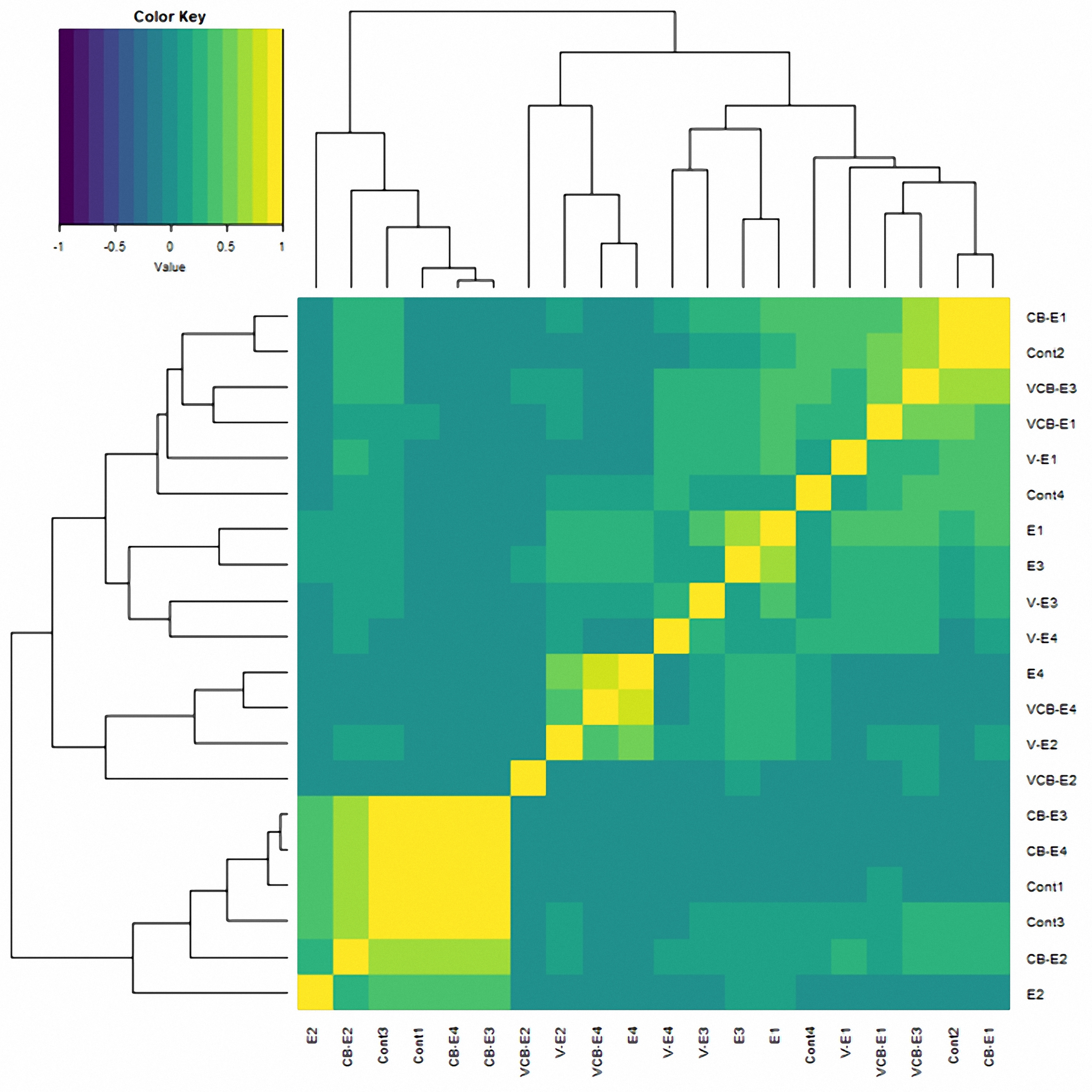

Supplement: Supplementary Figure S5 — Correlation heatmap for the intestine microbiome from samples pretreated with Clostridium butyricum and coccidiosis vaccine and then challenged with Eimeria sp. Correlation heatmap shows sample similarity by clustering the samples with similar taxa. Yellow indicates a close correlation, while green indicates a more distant correlation in the data. The heatmap was produced using the heatmap.2 function from gplots package in R version 4.1.0. [file Image_5.JPEG]
